# Rising mortality among people who inject drugs living with HIV in Scotland, UK: A 20‐year retrospective cohort study

**DOI:** 10.1111/hiv.13733

**Published:** 2024-11-18

**Authors:** R. Metcalfe, R. Fraser, K. M. A. Trayner, M. Glancy, A. Yeung, L. Sills, T. Ritchie, S. Priyadarshi, S. E. Peters, A. McAuley, S. Hutchinson

**Affiliations:** ^1^ School of Health and Life Sciences Glasgow Caledonian University Glasgow UK; ^2^ NHS Greater Glasgow & Clyde Glasgow UK; ^3^ Public Health Scotland, UK Glasgow UK

**Keywords:** drug‐related death, HIV, mortality, people who inject drugs

## Abstract

**Objectives:**

Our aim was to examine mortality trends in the era of antiretroviral therapy, among people who inject drugs (PWID) who are living with HIV. The study objectives were to assess and quantify mortality among PWID diagnosed with HIV over time in Scotland, in the context of a recent outbreak of HIV and rise in drug‐related mortality.

**Methods:**

This was a retrospective cohort study of those diagnosed with HIV in Scotland between January 2000 and February 2020, with acquisition related to injecting drug use, linked to mortality data. Factors associated with all‐cause mortality were examined using Cox proportional hazards regression.

**Results:**

Among 430 individuals with 3143 person‐years (py) of follow‐up, 88 (20.5%) died. Drug‐related deaths accounted for 45.5% of all deaths, rising to 60% among those diagnosed in 2015–2020. The crude all‐cause mortality was 28.00 per 1000 py overall and 37.62 per 1000 py within 5 years of diagnosis. Mortality risk was markedly higher among PWID diagnosed in 2015–2020 [adjusted hazard ratio (aHR) = 3.53], relative to those diagnosed in 2000–2004. Among those diagnosed in 2015–2020 (as part of the HIV outbreak), the mortality risk was higher among those not on, compared with those on, opioid agonist therapy (aHR = 3.87).

**Conclusion:**

Mortality among PWID living with HIV in Scotland has risen substantially in the 21st century. Our findings highlight the important role of opioid‐agonist therapy, alongside other prevention and treatment measures to address high levels of drug‐related mortality for PWID living with HIV, including within HIV outbreaks in this population group.

## INTRODUCTION

Mortality in people living with HIV (PLWH) is declining, with improvements in availability of antiretroviral therapy (ART) leading to increased rates of viral suppression, increasing HIV testing and efforts to engage people in lifelong care, resulting in life expectancy similar to the general population [1]. However, findings from a recent collaborative observational cohort study in Europe and North America found that among people who inject drugs (PWID) living with HIV, all‐cause mortality (ACM) declines were minimal in men (4% decrease over time), and for women the mortality rate increased by 7% [2] between 1996 and 2020.

People who inject drugs (PWID) are at increased risk of HIV acquisition through sharing of injecting equipment and sexual transmission [3, 4]. In 2013, a systematic review [5] demonstrated that among PWID, the ACM rate for those living with HIV was three times higher than for PWID who are HIV‐negative [5]. This increased mortality rate is not explained by HIV‐related mortality alone, with the study showing no difference in mortality rates from drug overdose and AIDS in this group [5]. The same authors demonstrated that non‐HIV/AIDS‐related mortality rate in PWID living with HIV in high‐income settings was 2.34 per 100 person‐years (py); more than 1.5 times higher than in PWID who are HIV‐negative [6]. Furthermore, PWID living with HIV have double the drug‐related mortality rate than those who are HIV‐negative [6]. Additionally, in 2024, Trickey et al. [2] reported that drug‐related mortality had increased over time in PLWH in North America, but decreased in Europe. Although there was an overall cohort reduction (adjusted cause‐specific mortality rate ratio = 0.94) the rates fluctuated over the 25‐year study period [2]. These data suggest that PWID living with HIV are at risk of increased mortality that is not mediated entirely through HIV/AIDS, and thus other interventions in addition to ART are required to reduce harm in this population group.

One of the largest and most persistent recent HIV outbreaks among PWID in the UK was recorded in Glasgow, Scotland's largest city [3]. Since 2015, prevalence of HIV among PWID in Glasgow City has risen more than 10‐fold, associated with homelessness, a rise in cocaine injecting as well as injecting in public places [4, 7], with approximately 200 new diagnoses (at the time of writing). The public health response to this HIV outbreak has included efforts to increase access to harm reduction services, scale‐up of HIV testing in key services, an enhanced model of outreach HIV care, extended partner notification and the dispensing of ART alongside opioid‐agonist therapy (OAT) via community pharmacies [8, 9].

Concurrently, drug‐related deaths in Scotland have increased rapidly since 2014 to one of the highest rates internationally [10]. Glasgow has one of the highest drug‐related death (DRD) rates in Scotland [11] and reviewing the interactions between both of these ongoing public health crises is fundamental to inform responses to prevent morbidity and mortality among PWID in Scotland. DRD in Scotland include those where the underlying cause of death was due to psychoactive substance use poisoning and where a drug listed under the Misuse of Drugs Act (1971) was known to be present in the body at the time of death [11].

The rise in drug‐related harms has occurred despite widespread access to the evidence‐based harm reduction interventions in Scotland [12, 13], including needle and syringe programmes (NSPs), OAT and widening access to naloxone. Other interventions, such as safe consumption facilities and drug‐checking services, are also recommended in international guidance [14], but have yet to be implemented.

For PWID living with HIV, adherence to ART is key to reducing the risk of HIV‐related morbidity and mortality [1]. Adapted HIV care service models in Glasgow have successfully supported high levels of ART adherence and viral suppression in PWID diagnosed during the HIV outbreak [8]. This is in line with recommendations since 2015 of ART for all PLWH, regardless of stage of infection, combined with retention in care, to reduce all‐cause morbidity and mortality [15, 16].

The aim of our study was to examine mortality trends in the era of ART, specifically among PWID, and during an outbreak of HIV. The study objectives were to assess and quantify mortality among PWID diagnosed with HIV over time in Scotland, with a particular focus on PWID diagnosed as part of the HIV outbreak in Glasgow.

## METHODS

### Study design, data sources and study cohort

We conducted a retrospective cohort study of those living with HIV in Scotland diagnosed between January 2000 and February 2020 (relating to the pre‐COVID period). Inclusion criteria for cohort entry was a presumed route of HIV acquisition related to injecting drug use. This route of HIV acquisition is self‐reported by the person at diagnosis and therefore includes all those who reported injecting drug use, including men who have sex with men (MSM). Individuals were identified within the Public Health Scotland (PHS) national HIV diagnosis database, which contains a record of all individuals diagnosed with HIV in Scotland since 1988 [17]. All individuals who receive healthcare in Scotland have a unique identifier known as the Community Health Index (CHI) number [18]. Using CHI, the HIV diagnosis database was linked to National Records of Scotland (NRS) death records (Scotland's national death registry) to obtain mortality information, including date and cause of death. To obtain data on hepatitis C virus (HCV) coinfection, HIV diagnosis records were further linked to national records for HCV testing and diagnosis held at PHS [19].

HIV diagnoses, for those with injecting drug use as a risk factor, were also linked to the Scottish National Prescribing Information System (PIS) which contains a record of all drugs which are paid for, prescribed and dispensed in the community in Scotland [20]. Data were extracted relating to the prescription of OAT for opioid dependence (i.e. methadone, buprenorphine and buprenorphine/naloxone) since 2010. To define the exposure, as per other studies [10], the date of reimbursement was used to determine periods on/off OAT (Supplementary File S1).

Approval for record linkage of UK National Health Service (NHS) data was provided by the NHS Public Benefit and Privacy Panel for Health and Social Care (PBPP 2021–0203).

### Outcomes and exposures

The primary outcome measure for this study was all‐cause mortality (ACM). Secondary outcome measures included drug‐related deaths and HIV‐related deaths. International Classification of Disease‐10 (ICD‐10) codes for underlying causes of death are described in Supplementary File S1, Table 1.

Exposure variables included: years since diagnosis (0–5 years/6–10/11+ years); age (continuous); sex (male/female); OAT status (on or off; time varying); time period of diagnosis (2000–2004/2005–2009/2010–2014/2015–2020); HCV status (never diagnosed/diagnosed with anti‐HCV antibodies).

### Statistical analysis

#### Factors associated with mortality among PWID diagnosed with HIV in Scotland (2000–2020)

Mortality rates were quantified for the analysis period 1 January 2000 through to 29 February 2020. Persons‐years was calculated from date of HIV diagnosis to the earliest of either date of death or 29 February 2020. We first estimated crude mortality rates overall and stratified by key demographics and comorbidities: calendar period of diagnosis, sex, age and HCV status.

Cause of death (DRD/HIV/other) by calendar period of HIV diagnosis, censored at 5 years since diagnosis, was reported as there was limited follow‐up time for those more recently diagnosed.

We fitted univariate and multivariate Cox proportional hazard regression models to estimate unadjusted and adjusted hazard ratios, respectively, of ACM associated with a parsimonious set of key covariates covering era, demographics and factors known to increase risk of mortality among PLWH: time period of diagnosis, sex, age and HCV status (diagnosed/undiagnosed).

To address the issue of longer follow‐up for those diagnosed earlier in the study period, we conducted a sensitivity analysis, censoring follow‐up time at 5 years after diagnosis. HCV status was not included in the sensitivity analysis due to lack of statistical power.

In supplementary analysis, we replaced the main exposure variable (calendar period of diagnosis), with a ‘years since diagnosis’ variable (0–5/6+ years) and confined analysis to those diagnosed prior to 2015, therefore excluding those with less than 5 years follow‐up available. This was to further assess if there was evidence of a high‐risk period of mortality shortly after diagnosis with HIV, which may be due to late diagnosis of HIV or other drug‐related harms around the time of HIV diagnosis.

Furthermore, we calculated crude mortality rates and univariate hazard ratios, using Cox proportional hazard regression models, for both drug‐related and HIV‐related deaths, by calendar period of HIV diagnosis, to determine if trends were driven by one specific cause of death.

We also calculated the rates of ACM by calendar time period to illustrate when the deaths occurred.

#### Factors associated with mortality among PWID diagnosed with HIV in Glasgow (during the HIV outbreak period 2015–2020)

To assess factors associated with mortality within the outbreak cohort in Glasgow, we restricted follow‐up time to begin on 1 January 2015 (when the outbreak was first identified) and included those diagnosed within the regional NHS Board (NHS Greater Glasgow & Clyde). In multivariable analysis we considered OAT exposure (on OAT vs. off OAT; time‐varying), sex and age.

## RESULTS

### Cohort characteristics and mortality

The cohort consisted of 430 individuals who were diagnosed with HIV in Scotland between 1 January 2000 and 29 February 2020 with 3143 py of follow‐up. Approximately two‐thirds (62.6%) were male and most (83%) were aged 45 or under at the time of diagnosis (Table 1).

**TABLE 1:** Characteristics of people who inject drugs who were diagnosed with HIV infection in Scotland during 2000–2020.^a^

Characteristic		*N* (%)
All		430 (100.0%)
Sex	Female	161 (37.44%)
	Male	269 (62.56%)
Age group (years)^b^	<35	183 (42.56%)
	36–45	173 (40.23%)
	>45	74 (17.21%)
Time period^b^	2000–2004	89 (20.70%)
	2005–2009	69 (16.05%)
	2010–2014	85 (19.77%)
	2015–2020^a^	187 (43.49%)
Time since HIV diagnosis^a^	0–5 years	236 (54.88%)
	6–10 years	75 (17.44%)
	11 + years	119 (27.67%)
CD4 count (cells/μL)^b^	Low <500	266 (61.86%)
	High >500	122 (28·37%)
	Missing	42 (9.77%)
Region^b^	NHS Greater Glasgow and Clyde board area	222 (51.63%)
	Rest of Scotland	208 (48.37%)
HCV status^b^	Not diagnosed	135 (31·40%)
	Diagnosed with anti‐HCV (not chronic)	295 (68.60%)
Deaths by cause	All‐cause	88 (20.47%)
	Drug‐related	40 (9.30%)
	HIV‐related	12 (2.79%)
	Liver disease/liver cancer	9 (2.09%)
	Non‐communicable diseases (cancer, heart disease, lung disease)	12 (2.79%)
	Suicide (including undetermined intent)	6 (1.40%)
	Other causes	9 (2.09%)

Abbreviations: HCV, hepatitis C virus.

^a^ Follow‐up period ends on 29 Feb 2020.

^b^ At time of HIV diagnosis.

There were 88 deaths within the study period, accounting for 20.5% of the overall cohort (Table 1). Drug‐related deaths (DRDs) accounted for nearly half (45.5%) of all deaths, with HIV‐related deaths accounting for 13.6% (Table 1).

**FIGURE 1:**
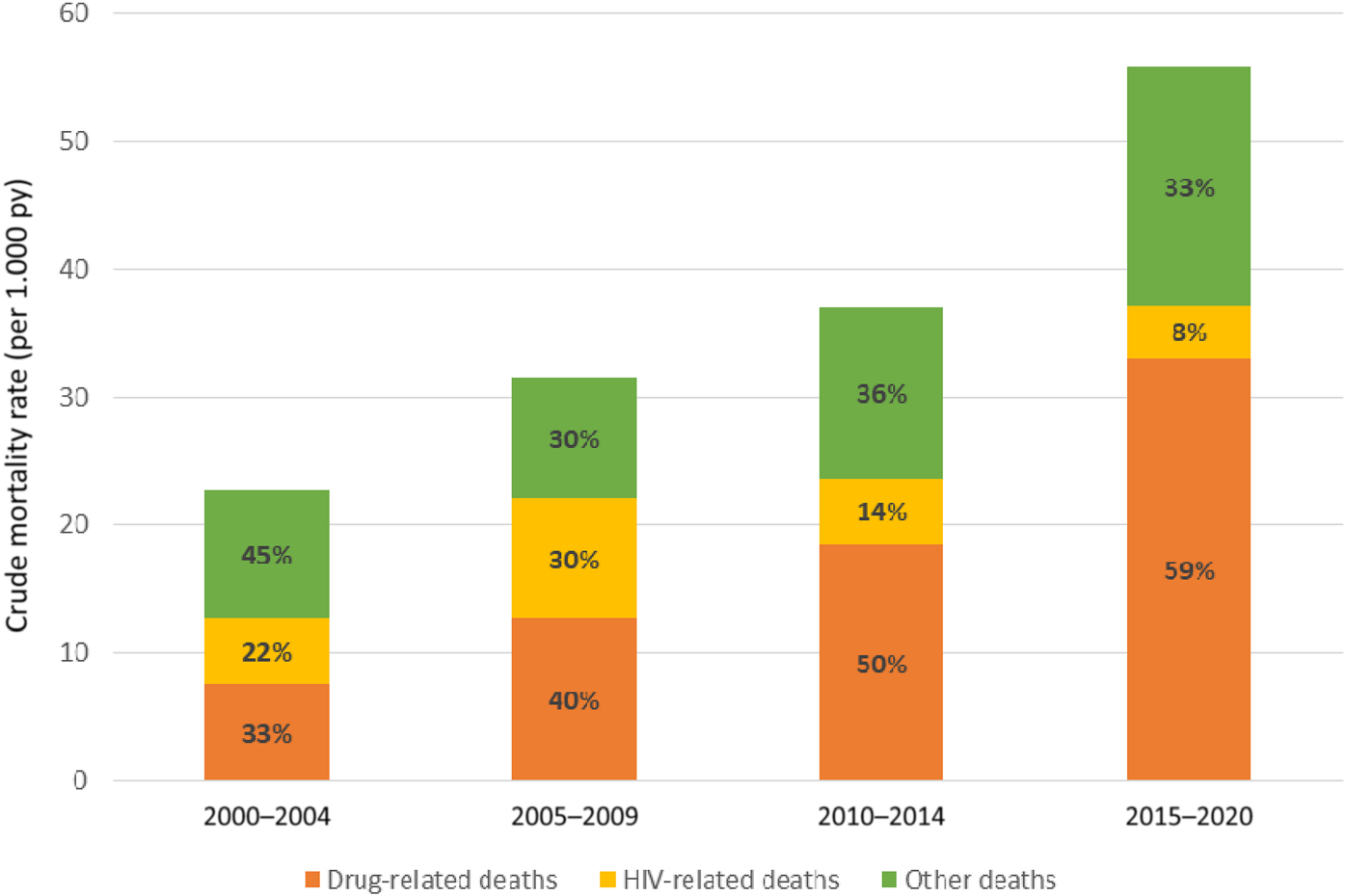
Crude mortality rates by period of HIV diagnosis, censored at 5 years after diagnosis.

When observing underlying causes of death by calendar period of diagnosis, censored at 5 years of follow‐up, the proportion of DRDs increased from approximately 33% (three deaths) in those diagnosed in 2000–2004, to around 60% (16 deaths) of the total deaths of those diagnosed in 2015–2020 (Figure 1). The overall crude mortality rate (CMR) in the cohort was 28.00 [95% confidence interval (CI): 22.70–34.51] per 1000 py (Table 2).

**TABLE 2:** Factors associated with all‐cause mortality among HIV‐diagnosed people who inject drugs (2000–2020^a^).

		Deaths (*n*)	Person‐years (py)	Mortality rate per 1000 py (95% CI)	Unadjusted hazard ratio^b^ (95% CI)	*p*‐value	Adjusted hazard ratio^b^, ^c^ (95% CI)	*p*‐value
Overall		88	3142.7	28.00 (22.72–34.51)				
Time period of HIV diagnosis	2000–2004	23	1361.5	16.89 (11.23–25.42)	1 (ref)		1 (ref)	
	2005–2009	19	745.6	25.48 (16.25–39.95)	1.87 (0.95–3.70)	0.072	1.60 (0.80–3.20)	0.181
	2010–2014	19	552.3	34.40 (21.94–53.93)	2.88 (1.36–6.09)	0.006	2.22 (1.03–4.79)	0.043
	2015–2020^a^	27	483.1	55.89 (38.33–81.49)	4.96 (2.23–11.00)	<0.001	3.53 (1.55–8.03)	0.003
Age (continuous)					1.03 (1.01–1.06)	0.010	1.02 (0.99–1.05)	0.145
Sex	Male	57	1822.7	31.27 (24.12–40.54)	1 (ref)		1 (ref)	
	Female	31	1319.9	23.49 (16.52–33.40)	0.75 (0.48–1.16)	0.195	0.93 (0.59–1.45)	0.736
HCV status (time‐dependent)	Not diagnosed	12	1010.6	11.87 (6.74–20.91)	1 (ref)		1 (ref)	
	Diagnosed	76	2132.1	35.65 (28.47–44.63)	2.88 (1.56–5.33)	0.001	2.28 (1.21–4.30)	0.011

Abbreviations: CI, confidence interval; HCV, hepatitis C virus.

^a^ Follow‐up period ends on 29 February 2020.

^b^ Modelled using Cox proportional hazard models.

^c^ Adjusted for age, sex and HCV status.

#### Factors associated with mortality among PWID diagnosed with HIV in Scotland (2000–2020)

There was a substantial increase in mortality according to calendar period of diagnosis, with CMRs increasing from 16.89 (11.23–25.42) per 1000 py in those diagnosed during 2000–2004 to 55.89 (38.33–81.49) per 1000 py in those diagnosed during 2015–2020 (Table 2). In the multivariable model, this increasing trend over time remained after adjustment, with mortality risk in those diagnosed in 2015–2020 over three times greater [adjusted hazard ratio (aHR) = 3.53, 95% CI: 1.55–8.03] than in 2000–2004. Differences in mortality risk were less evident for both sex and age. For HCV status, being diagnosed with HCV had an increased risk of mortality compared with the never‐diagnosed group (aHR = 2.28, 95% CI: 1.21–4.30).

Supplementary analysis of ACM calculated by time period of death is shown in Supporting information, Supplementary File S3, Table 2. The number of deaths in each calendar time period increased over time but the mortality rate increase was not significant in the adjusted analysis.

For DRDs, there was evidence of an increasing trend of drug‐related mortality if diagnosed in a more recent calendar period, rising from 5.88 per 1000 py in 2000–2004 to 33.12 per 1000 py in 2015–2020 (HR = 4.84, *p* = 0.006). There was no evidence of an increase in HIV‐related deaths if diagnosed in a more recent calendar period, with these events much rarer (Table 3).

**TABLE 3:** Crude mortality rates and hazard ratios for drug‐related deaths, HIV‐related deaths and other deaths among people who inject drugs (PWID) living with HIV (2000–2020^b^).

	Drug‐related deaths					HIV‐related deaths					Other deaths				
	Number of deaths	PYFU	Mortality rate per 1000 PYFU (95% CI)	Unadjusted hazard ratio^a^ (95% CI)	*p*‐value	Number of deaths	PYFU	Mortality rate per 1000 PYFU (95% CI)	Unadjusted hazard ratio^a^ (95% CI)	*p*‐value	Number of deaths	PYFU	Mortality rate per 1000 PYFU (95% CI)	Unadjusted hazard ratio^a^ (95% CI)	*p*‐value
Overall	40	3142.7	12.73 (9.34–17.35)			12	3142.7	3.82 (2.17–6.72)			36	3142.7	11.46 (8.26–15.88)		
Time period of diagnosis															
2000–2004	8	1362	5.88 (2.94–11.75)	1 (ref)		2	1362	1.47 (0.37–5.87)	1 (ref)		13	1362	9.55 (5.54–16.44)	1 (ref)	
2005–2009	7	745.6	9.39 (4.48–19.69)	2.03 (0.69–5.99)	0.201	5	745.6	6.71 (2.79–16.11)	3.27 (0.64–16.87)	0.156	7	745.6	9.39 (4.48–19.69)	0.81 (0.32–2.05)	0.664
2010–2014	9	552.3	16.29 (8.48–31.32)	3.80 (1.16–12.50)	0.028	3	552.3	5.43 (1.75–16.84)	1.76 (0.29–10.56)	0.536	7	552.3	12.67 (6.04–26.58)	1.17 (0.43–3.14)	0.758
2015–2020^2^	16	483.1	33.12 (20.29–54.06)	4.84 (1.56–15.05)	0.006	2	483.1	4.14 (1.04–16.55)	1.04 (0.14–7.85)	0.971	9	483.1	18.63 (9.69–35.80)	1.70 (0.60–4.84)	0.322

Abbreviations: CI, confidence interval; PYFU, person years follow.

^a^ Modelled using Cox proportional hazard models.

^b^ Follow‐up ends on 29 February 2020.

In sensitivity analysis, after censoring the follow‐up time to 5 years post‐diagnosis, the trend of increasing mortality by calendar period of diagnosis was observed but we lack statistical power in multivariate analysis [HR = 2.25 (*p* = 0.038) and aHR = 1.88 (*p* = 0.114)] (Supporting information Supplementary File S2, Table 1).

In the supplementary analysis, the risk of mortality was substantially higher in the 0–5 year period after diagnosis (aHR = 1.54, 95% CI: 0.86‐2.76) compared with 6+ years since diagnosis (Supporting information, Supplementary File S3, Table 1).

### Factors associated with mortality during the HIV outbreak

The overall CMR for those diagnosed with HIV in Glasgow between 2015 and 2020 was 65.18 (95% CI: 43.69–97.24) per 1000 py. (Table 4). There was substantially higher CMR for individuals who were off OAT (129.24, 95% CI: 7.92–214.3 per 1000 py) compared with the on‐OAT group (36.43, 95% CI: 18.95–70.01 per 1000 py). After adjustment for age and sex, the protective effect of OAT remained, with mortality risk among those off OAT being nearly four times greater (aHR = 3.87, 95% CI: 1.66–9.00) than among those on OAT. Females were shown to have increased risk of mortality (aHR = 2.32, 95% CI: 1.02–5.27) compared with males.

**TABLE 4:** Factors associated with all‐cause mortality among people who inject drugs (PWID) diagnosed with HIV during the outbreak in Glasgow (2015–2020^a^).

		Deaths (*n*)	Person‐years (py)	Mortality rate per 1000 py (95% CI)	Unadjusted hazard ratio^a^ (95% CI)	*p*‐value	Adjusted hazard ratio^b^, ^c^ (95% CI)	*p*‐value
Overall		24	368.2	65.18 (43.69–97.24)				
OAT status (time‐varying)	On OAT	9	247.1	36.43 (18.95–70.01)	1 (ref)		1 (ref)	
	Off OAT	15	116.1	129.24 (77.92–214.38)	3.43 (1.49–7.87)	0.004	3.87 (1.66–9.00)	0.002
Age (continuous)					1.03 (0.97–1.09)	0.359	1.03 (0.98–1.09)	0.298
Sex	Male	12	239.4	50.13 (28.47–88.26)	1 (ref)		1 (ref)	
	Female	12	128.8	93.14 (52.90–164.01)	1.86 (0.84–4.15)	0.127	2.32 (1.02–5.27)	0.045

Abbreviations: CI, confidence interval; OAT, opioid‐agonist therapy.

^a^ Follow‐up period ends on 29 February 2020.

^b^ Modelled using Cox proportional hazard models.

^c^ Adjusted for age and sex.

## DISCUSSION

In the context of an HIV outbreak and high levels of drug‐related mortality, we quantified mortality among a cohort of individuals following their HIV diagnosis, with injecting drug use as the likely route of acquisition. We found that mortality among PWID living with HIV in Scotland has increased over a 20‐year period and was 3.5 times higher among those diagnosed in 2015–2020 (relating to the outbreak period) than among those diagnosed in the early study period at the turn of the century. This is supported by the adjusted analysis of mortality by time period of death not showing an increase.

However, OAT was strongly protective against ACM among those diagnosed during the outbreak period, associated with a 75% reduction in risk. This is a similar finding to other studies [4] and it should be acknowledged that whilst engaged with OAT, PWID are likely also to benefit from input from a range of other services, which may be protective.

Although sex was not a significant factor in the overall cohort, during the outbreak period, females were found to be an increased risk of mortality. This time period is recognized as an era of increased DRDs among women in Scotland with multiple possible contributing factors. [21].

There is limited research to date quantifying mortality trends and factors associated with mortality specifically in PWID living with HIV, and less reporting on this in the context of an HIV outbreak. Scotland is a country with moderate to high coverage of the recommended harm reduction interventions to reduce morbidity and mortality in PWID, with extensive, free‐to‐access OAT, NSPs and naloxone [22]. However, PWID need to access these services at levels that are high enough to prevent negative behaviours/risk associated with mortality, including DRD. In Glasgow, diamorphine‐assisted treatment was introduced towards the end of the study period [23]. For people living with HIV, ART is provided free of charge on the NHS and as soon as possible after diagnosis, in line with national treatment guidance [24] and services have adapted to a bespoke model of care for PWID [8].

Despite these interventions, our study demonstrates a worrying increase in drug‐related mortality over time, with 60% of deaths among PWID living with HIV in 2015–2020 deemed to be drug‐related. The overall CMR of 28 per 1000 py and peak CMR in 2015–2020 of 55.89 per 1000 py far exceed the pooled estimates reported in previous cohort studies (2.35 per 100 py [5], 11.1 per 1000 py) [2].

Drug‐related mortality accounted for nearly half of the deaths within the HIV outbreak cohort. Scotland's DRD rate increased rapidly during the outbreak period partly due to changing polydrug use patterns, including increased use of non‐prescribed (‘street’) benzodiazepines, gabapentinoids and injection of powder cocaine, alongside opioids [10, 25, 26]. To our knowledge, this is the first study to report on the specific cause of death within recent HIV outbreak cohorts of PWID. The only other recent study to date examining mortality among PWID in the context of an HIV outbreak estimated ACM among PWID in Athens, Greece [27]. They reported that HIV infection was associated with an increase in ACM and that PWID living with HIV had more than double (32.12) the standardized mortality ratio (SMR) than PWID who were HIV‐negative (14.00).

Despite high rates of DRDs, OAT was strongly protective against mortality during the outbreak and associated with a nearly four‐fold reduction in risk, supporting existing evidence [28, 29]. This finding may be expected given the wider evidence of OAT effectiveness in PWID, and our study contributes to this evidence in the specific context of an HIV outbreak – therefore demonstrating the importance of prioritizing OAT engagement and retention, alongside other key prevention intervention, such as NSPs, HIV testing and treatment, in the public health response to an HIV outbreak among PWID.

Our findings are in contrast to the improved survival overall for PLWH, due to widening access to ART and reduction in HIV/AIDS‐related deaths [1]. However, more recently an increase in drug‐related mortality among PLWH has been also observed in North America [2], where there is less OAT coverage, and for PWID living with HIV the authors reported an increase in mortality for women and a small decrease in men. These findings complement our study and demonstrate the importance of reporting on specific groups when examining mortality and causes in order to develop interventions to reduce mortality.

In Scotland, our findings demonstrate that policy‐makers must focus on further interventions to reduce drug‐related harms, including HIV. Although there is moderate to high coverage of harm reduction interventions [22] and standards for medication‐assisted treatment [30], further efforts are needed to enhance the prevention infrastructure, such as the introduction of safer drug consumption facilities and drug‐checking facilities for PWID; both of these interventions are scheduled for implementation in Scotland in 2024.

HIV prevention must be resourced within these structures as Scotland has committed to achieving HIV transmission elimination by 2030 [31] and therefore a focus on widespread and frequent HIV testing, and early linkage to HIV care or HIV prevention measures (e.g. HIV pre‐exposure prophylaxis) with adapted service models to suit this vulnerable group are crucial. There has already been progress in this area in Glasgow [8, 32] and there should be a commitment to continue this nationally to reduce HIV transmission and HIV‐related mortality in this group. The findings from the focused analysis on the outbreak cohort demonstrate the very high risk of mortality for those who acquired HIV during an outbreak, and that public health interventions must include prioritizing OAT, NSPs as well as treatment services for HIV and HCV.

For PWID living with HIV, it is reassuring that HIV‐related mortality is decreasing. However, in an era of ART for all and advances in ART, this cohort was still experiencing HIV‐related mortality in 2015–2020. ART should be accessible to all, but Scottish data have shown that ART availability to PWID has been slower than other groups of PLWH [33]. A recent scoping review [34] confirmed this variability in achievement of ART adherence and viral suppression in PWID living with HIV. It found that although HIV/AIDS‐related mortality is decreasing in PWID, as with other groups, a gap remains for this group, who may also be impacted by compounding coinfections, such as hepatitis B and C and skin and soft tissue infections. It is important to highlight that there is no indication that ART use in this cohort contributed to deaths, and in the HIV outbreak cohort, the ART of choice included integrase inhibitors to minimize potential drug interactions.

If reduction in HIV‐related morbidity and mortality, and reduction in transmission overall in line with national and international goals [31, 35] are to be achieved, it is important to understand more fully the mortality rate, and causes, among PWID living with HIV.

Furthermore, focusing on measures to reduce late HIV diagnosis and ensuring that treatment services can work with other key services to provide accessible ART for PWID in the early stages after HIV diagnosis are imperative, as we have shown the risk of mortality to be higher soon after diagnosis. This vulnerable group faces barriers to accessing traditional care models, especially due to stigma associated with injecting drug use [36]. HIV treatment and prevention services must be able to adapt to provide services for treatment and monitoring, which integrate with other drug‐related harm prevention services, in order to reduce these barriers [8].

Our study has a number of limitations. First, HIV acquisition risk is self‐reported by individuals via clinical services, and in some cases this is incomplete, which could lead to underreporting of numbers of PWID living with HIV in Scotland and exclusion from the study. Second, cause of death data are taken from death certificates and these are completed by the doctor who is certifying the death. The data were collated retrospectively and without full medical records, and therefore there is a possibility that inaccurate completion of the certificate could lead to HIV being coded as a direct cause of death rather than an indirect one. It is also possible that the HIV recording protocol on death certificates changed over the study time period, with potentially more recording of HIV in early time periods compared with later ones. The death certificates were not accessed in the study so it was not possible to adjust for this. Furthermore, in the sensitivity analysis, the trend of increasing mortality over the period of diagnosis, as seen in the primary model, diminished. This is probably due to the small numbers included in each time period in this analysis, resulting in a lack of statistical power.

## CONCLUSION

Mortality among PWID living with HIV in Scotland has risen substantially in the 21st century. Our findings highlight the urgent need to sustain a multi‐intervention response to address the high levels of mortality in Scotland, and particularly in Glasgow, the setting of the UK's largest HIV outbreak among PWID in 30 years. Without it, Scotland's pursuit of HIV transmission elimination and its ‘national mission’ to reduce DRDs will be challenging, and health inequalities are likely to be widened among this most marginalized of populations.

## AUTHOR CONTRIBUTIONS

RM, AMcA and SH designed the research idea. All authors contributed to data collection. RM, RF, KT, AMcA and SH interpreted the data and drafted the initial manuscript. All authors reviewed, made contributions and approved the final manuscript and all authors agree to be accountable for the work.

## CONFLICT OF INTEREST STATEMENT

None.

## Supporting information


**Data S1.** Supporting information.
