# Supplementary material for: Rising mortality among people who inject drugs living with HIV in Scotland, UK: A 20‐year retrospective cohort study
Source: HIV Med. 2024 Nov 18;26(2):265–74. doi: 10.1111/hiv.13733 (PMC11786617; doi:10.1111/hiv.13733)
Supplement: Supplementary file 1 — Data S1. Supporting information. [file HIV-26-265-s001.docx]

**Supplementary File S1**

***Data Sources***

**Exposure: Opioid agonist treatment (OAT)**

**The following information on the exposure variable was first reported in McAuley et al 2023.^1^**

From Scotland’s national Prescribing Information System (PIS) we obtained all prescriptions for methadone, buprenorphine and buprenorphine-naloxone, between 2010 and 2020.^2^ For each prescription record, we obtained the reimbursement date, prescription date (where available), health board of residence and type of medication.

Reimbursement dates are available for all prescriptions and are aggregated by month, with the date always falling at the end of the month. Payments are made when the prescription is fully dispensed, which creates a time-lag within the data: for example, a 28-day prescription made in mid-March may not be fully dispensed until mid-April and the date of reimbursement will therefore fall on 30 April. Prescription date is available only for a subset of records from electronic general practitioner prescriptions. In our sample, 43% of all records had a prescription date and the proportion of records with a prescription date has decreased over time from 57% in 2010 to 33% in 2020. Moreover, very few individuals overall (4.5%) had a complete record of prescription dates throughout the study period. We therefore used the date of reimbursement to determine periods on/off OAT for consistency.

For the 43% of prescriptions with a prescription date, the average (mean) number of days between date of prescription and reimbursement was 40 (SD, 20), consistent with previous analysis.^3^ For the subset of our sample with a complete record of prescription dates, the median number of days between consecutive prescription dates was 28 (IQR, 14 to 28), which is consistent with clinical guidelines on ‘good practice’ for length of prescriptions in the UK.^4^

Using these data, we estimated that a prescription date is likely to fall at any point in the 60 days before the date of reimbursement (based on the time-lag described above) and be fully dispensed by 12 days before the date of reimbursement (based on an average of 40 days between prescription and reimbursement, with a prescription length of 28 days (as informed by available data, above)). We therefore coded new treatment episodes as commencing 60 days before the first date of reimbursement and ending 12 days before the last date of reimbursement. We defined a continuous episode of treatment as a pattern of regular monthly dates of reimbursement with no more than two months between them. As prescription data may be incomplete in longer treatment episodes and prescriptions may span two months (as described above), we hypothesized that a shorter period (i.e., one missed monthly reimbursement) may not be indicative of a gap in treatment. All remaining follow up time was coded as 'off' treatment.

We included in our study individuals who received at least one prescription between 2015 and 2020. The start of follow-up was defined as the first estimated date of prescription.

Table 1s: Description and ICD-10 codes of underlying causes of death.

| **Description of underlying cause of death** | **ICD-10 Codes** |
| --- | --- |
| *Drug-related death^5^* |  |
| Mental and behavioural disorder due to psychoactive drug use (excluding alcohol and tobacco)  Accidental poisoning by and exposure to noxious substances  Intentional self-harm  Assault by drugs, medicaments, and biological substances  Poisoning by undetermined intent | F11, F12, F13, F14, F15, F16, F19 |
|  | X40, X41, X42, X43, X44 |
|  | X60, X61, X62, X63, X64 |
|  | X85 |
|  | Y11, Y12, Y13, Y14 |
| *HIV-related death* | B20, B21, B22, B23, B24 |
| *Hepatitis C, Liver disease and liver cancer* |  |
| Viral hepatitis  Diseases of the liver  Liver cancer | B15, B16, B17, B18, B19  K70, K71, K72, K73, K74, K75, K76, K77  C22 |
| *Non-communicable diseases (heart disease, lung disease, cancers)*  Ischaemic heart disease  Pulmonary heart disease and diseases of the Pulmonary circulation  Other forms of heart disease  Chronic lower respiratory diseases  Malignant neoplasms of respiratory and intrathoracic organs | I20, I21, I22, I23, I24, I25  I26, I27, I28  I30, I31, I32, I33, I34, I35, I36, I37, I38, I39, I40, I41, I42, I43, I44, I45, I46, I47, I48, I49, I50, I51, I52  J40, J41, J42, J43, J44, J45, J46, J47  C30, C31, C32, C33, C34, C35, C36, C37, C38, C39 |
| *Suicide*  Intentional self-harm by drugs  Intentional self-harm by other means  Undetermined Intent  Sequelae of intentional self-harm  Sequelae of events of undetermined intent | X60, X61, X62, X63, X64  X65, X66, X67, X68, X69, X70, X71, X72, X73, X74, X75, X76, X77, X78, X79, X80, X81, X82, X83, X84  Y10, Y11, Y12, Y13, Y14, Y15, Y16, Y17, Y18, Y19, Y20, Y21, Y22, Y23, Y24, Y25, Y26, Y27, Y28, Y29, Y30, Y31, Y32, Y33, Y34  Y87.0  Y87.2 |

References:

1. McAuley A, Fraser R, Glancy M, et. al. Mortality among individuals prescribed opioid-agonist therapy in Scotland, UK, 2011-20: a national retrospective cohort study. *Lancet Public Health* 2023; **8:** e484-e493.

2. Alvarez-Madrazo S, McTaggart S, Nangle C, Nicholson E, Bennie M. Data Resource Profile: The Scottish National Prescribing Information System (PIS). *Int J Epidemiol* 2016; **45:** 714-715f.

3. Gao L, Dimitropoulou P, Robertson JR, McTaggart S, Bennie M, Bird SM. Risk-factors for methadone-specific deaths in Scotland's methadone-prescription clients between 2009 and 2013. *Drug Alcohol Depend* 2016; **167:** 214‐223.

4. Clinical Guidelines on Drug Misuse and Dependence Update 2017 Independent Expert Working Group. Drug misuse and dependence: UK guidelines on clinical management. 2017. <https://assets.publishing.service.gov.uk/government/uploads/system/uploads/attachment_data/file/673978/clinical_guidelines_2017.pdf>

5. National Records of Scotland (NRS). Drug-related deaths in Scotland in 2022. August 22, 2023. [Drug-related Deaths in Scotland in 2022 - Report (nrscotland.gov.uk)](https://www.nrscotland.gov.uk/files/statistics/drug-related-deaths/22/drug-related-deaths-22-report.pdf) (accessed Nov 15, 2023).

**Supplementary File S2**

***SENSITIVITY ANALYSIS***

Table 1: Trends in all-cause mortality over time, among HIV positive PWID (2000-2020^2^): censoring follow-up to 5 years post diagnosis.

|  | **number of deaths** | **person years (py)** | **mortality rate per 1000 PYFU (95% CI)** | **Unadjusted Hazard ratio^1^**  **(95% CI)** | **p-value** | **Adjusted Hazard ratio^1,3^**  **(95% CI)** | **p-value** |
| --- | --- | --- | --- | --- | --- | --- | --- |
| **Overall** | **60** | **1,594.9** | **37.62 (29.21, 48.45)** |  |  |  |  |
| **Time Period of Diagnosis** |  |  |  |  |  |  |  |
| **2000-2004** | 9 | 415.0 | 21.69 (11.28, 41.68) | 1 (ref) |  | 1 (ref) |  |
| **2005-2009** | 10 | 317.6 | 31.49 (16.94, 58.52) | 1.45 (0.59, 3.57) | 0.419 | 1.29 (0.42, 3.20) | 0.589 |
| **2010-2014** | 14 | 379.5 | 36.89 (21.85, 62.29) | 1.70 (0.73, 3.92) | 0.215 | 1.44 (0.61, 3.39) | 0.405 |
| **2015-2020^2^** | 27 | 482.8 | 55.92 (38.35, 81.54) | 2.25 (1.05, 4.83) | 0.038 | 1.88 (0.86, 4.14) | 0.114 |
| **Age (continuous)** |  |  |  | 1.04 (1.01, 1.07) | 0.018 | 1.03 (1.00, 1.06) | 0.079 |
| **Sex** |  |  |  |  |  |  |  |
| **Male** | 40 | 968.6 | 41.30 (30.29, 56.30) | 1 (ref) |  | 1 (ref) |  |
| **Female** | 20 | 626.3 | 31.93 (20.60, 49.50) | 0.79 (0.46, 1.35) | 0.386 | 0.88 (0.51, 1.52) | 0.647 |

^1^Modelled using cox proportional hazard models. ^2^Follow-up ends on 29^th^ Feb 2020. ^3^Adjusted for age and sex.

**Supplementary File S3**

***ADDITIONAL SUPPLEMENTARY ANALYSES***

Table 1: Trends in all-cause mortality over time, among HIV positive PWID (2000-2014^2^)

|  | **number of deaths** | **person years (py)** | **mortality rate per 1000 PYFU (95% CI)** | **Unadjusted Hazard ratio^1^**  **(95% CI)** | **p-value** | **Adjusted Hazard ratio^1^**  **(95% CI)** | **p-value** |
| --- | --- | --- | --- | --- | --- | --- | --- |
| **Overall** | **61** | **2,659.5** | **22.94 (17.85, 29.48)** |  | | | |
| **Years since diagnosis** |  |  |  |  |  |  |  |
| **0-5 years** | 37 | 1,315.8 | 28.12 (20.37, 38.81) | 1.69 (0.95, 3.03) | 0.077 | 1.54 (0.86, 2.76) | 0.145 |
| **6+ years** | 24 | 1,343.7 | 17.86 (11.97, 26.65) | 1 (ref) |  | 1 (ref) |  |
| **Age (continuous)** |  |  |  |  |  |  |  |
|  |  |  |  | 1.03 (1.00, 1.06) | 0.055 | 1.02 (0.98, 1.05) | 0.322 |
| **Sex** |  |  |  |  |  |  |  |
| **Male** | 44 | 1,511.5 | 29.11 (21.66, 39.12) | 1 (ref) |  | 1 (ref) |  |
| **Female** | 17 | 1,148.0 | 14.81 (9.21, 23.82) | 0.50 (0.29, 0.88) | 0.017 | 0.62 (0.35, 1.11) | 0.106 |
| **HCV status (time-varying)** |  |  |  |  |  |  |  |
| **Not Diagnosed** | 10 | 974.5 | 10.26 (5.52, 19.07) | 1 (ref) |  | 1 (ref) |  |
| **Diagnosed** | 51 | 1,685.1 | 30.27 (23.00, 39.82) | 2.99 (1.51, 5.90) | 0.002 | 2.57 (1.29, 5.12) | 0.008 |

^1^Modelled using cox proportional hazard models. ^2^Follow-up ends on 29^th^ Feb 2020. ^3^Adjusted for age, sex and HCV status.

Table 2: Factors associated with all-cause mortality among HIV diagnosed PWID (2000-2020^2^) by calendar time period

|  | | **number of deaths** | **Person years (pys)** | **Mortality rate per 1000 pys (95% CI)** | **Unadjusted Hazard Ratio^1^ (95% CI)** | **P-value** | **Adjusted Hazard Ratio^1,3^ (95% CI)** | **P-value** |
| --- | --- | --- | --- | --- | --- | --- | --- | --- |
| **Overall** | | 88 | 3,142.7 | 28.00 (22.72, 34.51) |  | | | |
| **Time period** | 2000 – 2004 | 6 | 241.9 | 24.80 (11.14, 55.21) | 1 (ref) |  | 1 (ref) |  |
|  | 2005 – 2009 | 12 | 572.3 | 20.97 (11.91, 36.92) | 1.24 (0.45, 3.36) | 0.679 | 1.10 (0.40, 3.00) | 0.851 |
|  | 2010 – 2014 | 23 | 853.5 | 26.95 (17.91, 40.55) | 1.79 (0.71, 4.51) | 0.219 | 1.38 (0.54, 3.52) | 0.500 |
|  | 2015 – 2020^2^ | 47 | 1,475.0 | 31.86 (23.94, 42.41) | 2.09 (0.88, 4.96) | 0.096 | 1.43 (0.59, 3.47) | 0.427 |

^1^ Modelled using cox proportional hazard models. ^2^ Follow-up period ends on 29^th^ Feb 2020. ^3^Adjusted for age, sex and HCV status.
